# Prevalence and associated factors of hypertension among adult patients attending the outpatient department at the primary hospitals of Wolkait tegedie zone, Northwest Ethiopia

**DOI:** 10.3389/fneur.2022.943595

**Published:** 2022-08-09

**Authors:** Daniel Gashaneh Belay, Haileab Fekadu Wolde, Meseret Derbew Molla, Hailu Aragie, Dagnew Getnet Adugna, Endalkachew Belayneh Melese, Gebrekidan Ewnetu Tarekegn, Eleleta Gezahegn, Anteneh Ayelign Kibret

**Affiliations:** ^1^Department of Human Anatomy, School of Medicine, College of Medicine and Health Sciences, University of Gondar, Gondar, Ethiopia; ^2^Department of Epidemiology and Biostatistics, College of Medicine and Health Sciences, Institute of Public Health, University of Gondar, Gondar, Ethiopia; ^3^Department of Biochemistry, School of Medicine, College of Medicine and Health Sciences, University of Gondar, Gondar, Ethiopia; ^4^Department of Internal Medicine School of Medicine, College of Medicine and Health Sciences, University of Gondar, Gondar, Ethiopia

**Keywords:** hidden hypertension, associated factors, Ethiopia, wolkite, war

## Abstract

**Introduction:**

Hypertension, often known as increased blood pressure, is a worldwide public health concern. Globally, ~1 billion people have hypertension and 7.1 million die from this disease. It is disproportionately more prevalent in resource-poor nations, with inadequate health systems like Ethiopia. Moreover, information on the burden of disease from hypertension in the specific area, especially in the newly organized Wolkait Tegedie zone, is essential to develop effective prevention and control strategies. Therefore, this study aimed to assess the prevalence of hypertension and associated factors among adult patients evaluated at the outpatient department of the two district hospitals in the Wolkait Tegedie zone, Northwest Ethiopia.

**Methods:**

An institution-based cross-sectional study was conducted from September to October 2021. A systematic random sampling technique was used to select a total of 449 participants. The data were collected and then entered using EPI-INFO version 7 and exported to STATA 14 for analysis. Bivariable and multivariable binary logistic regression analyses were performed. Adjusted odds ratio (AOR) with a 95% confidence interval (CI) was used as a measure of association. Variables having a *p*-value < 0.05 from the multivariable analysis were considered to have a significant association with the outcome.

**Results:**

The prevalence of hypertension among adult patients in this study was 44.91% [95% CI: 40.26%, 49.65%], of which 63.92% were new diagnoses. Being >60 years [AOR = 1.81; 95% CI: 1.11, 3.20], having poor physical exercise [AOR = 1.74; 95% CI: 1.01, 3.15], consuming cruddy oil [AOR = 15.22; 95% CI: 3.86, 60.12], having a family history of hypertension [AOR = 13.02; 95% CI: 3.75, 45.16], and having a history of diabetes mellitus (DM) [AOR = 8.05; 95% CI: 1.24, 51.49] had a statistically significant association with having hypertension.

**Conclusion:**

There is a relatively high prevalence of hypertension among adult patients in the outpatient department of the two primary hospitals, Northwest Ethiopia. Factors such as being of older age, having poor physical exercise behavior, cruddy oil consumption, and family history of DM and hypertension had a positive significant statistical association with being hypertensive. Community-based screening programs for hypertension should be designed and implemented to prevent this silent killer disease. Health education and promotion that focus on healthy nutrition and physical exercise should be delivered.

## Introduction

Globally, non-communicable diseases (NCDs) account for approximately three-fourths of all deaths (1, 2). Hypertension, often known as increased blood pressure, is a worldwide public health concern (1). Globally, ~1.4 billion people have hypertension (2). Its burden is also projected to be 1.56 billion worldwide in 2025, two-thirds of which is expected to occur in the developing countries (3, 4).

In low socioeconomic countries, in addition to the big challenge of curing communicable diseases such as malaria and HIV, the increasing problem of hypertension in this region makes a double burden and a serious consequence (5). In resource-poor nations with inadequate health systems, this disease is disproportionately more prevalent (1). Many people go undiagnosed because hypertension rarely shows symptoms in the early stages (1). It is usually diagnosed incidentally or after major organ damage has occurred (6). Even those who are diagnosed may not have access to treatment and may not be able to maintain long-term management of their sickness (1).

Increased blood pressure is one of the principal risks of life-threatening complications on vital organs such as the heart, blood vessels, kidneys, and brain, which leads to premature disability and mortality (7). Globally, ~49% of ischemic heart diseases and 62% of cerebrovascular diseases are attributable to hypertension that could have been reduced by taking antihypertensive drugs (8). In Africa, hypertension was thought to be rare, but now it is known as one of the most important cerebrovascular diseases, contributing to ~40% of these diseases on the continent (6). In Ethiopia, the epidemiology of hypertension is not well studied. But some studies showed that its prevalence ranges from 1.8% in rural communities (9) to 30% in urban regions (10). While according to the national NCDs STEPS survey, the overall prevalence of hypertension was 15% (11).

Studies showed that too much salt and fat consumption (1, 6), alcohol use (1, 6), physical inactivity, and lack of exercise (1) were considered the risk factors for hypertension. It is also common among patients with advanced age and patients who live in urban residences (6).

Treating the complications of hypertension such as cardiac bypass surgery, stroke management, and dialysis is a costly intervention that drains individual and government budget (1). Significant health and economic gains are attached to early detection and good control of hypertension (12). Information on the burden of disease from hypertension in a specific area is essential in developing effective prevention and control strategies. Therefore, this study aimed to assess the prevalence of hypertension and associated factors among adult patients evaluated in the outpatient department (OPD) for different reasons in the Kiraker and Nigus Ketema primary hospitals, Northwest Ethiopia. The result of this study may contribute to the hospital as well as to the country in drawing the attention of the policymakers, healthcare managers, and healthcare professionals to have early detection and good control of hypertension.

## Methodology

### Study design and period

An institution-based cross-sectional study was conducted from 1 September to 30 October, 2021, among patients who come to the OPD at the two primary hospitals in the Wolkait Tegedie zone. The Wolkait Tegedie zone is a newly emerging and newly organized zone in the Amhara region formed in 2021. Currently, it has 5 primary hospitals and 19 health centers. From these, ~13,000 and 8,000 patients are expected to visit adult OPD per year in the Kiraker and Nigus Ketema primary hospitals, respectively.

### Source and study population

The source population for this study was all the adult patients who come to the OPD of the two primary hospitals. Those patients who were available at the time of data collection were considered as the study population. However, adult patients who were unable to communicate and those with severe psychiatric problems were excluded from the study.

### Sample size and sampling procedure

The sample size for studying the prevalence of hypertension and associated factors was determined using both the precision approach and the power approach. The precision approach was calculated using a single-population proportion formula by considering a prevalence of 28.3% from the study done in the nearest setting (13), 95% confidence interval (CI), and 5% margin of error. Therefore, using Cochran's sample size formula (Table 1):


(1) $$\begin{array}{r} \text{n}=\frac{{\left({\text{Z}}_{\text{a}/2}\right)}^{2}\text{pq}}{{\text{d}}^{2}},\text{ n}=\frac{{\left(1.96\right)}^{2}\left(0.283\times \;0.717\right)=312}{{\left(0.05\right)}^{2}} \end{array}$$

Therefore, from the above alternatives, the highest sample size means the sample derived based on the power approach Kelsey method for variable underweight was used (*n* = 449).

**Table 1:** Sample size calculation based on the two objectives.

**I. Based on a precision approach (single population proportion)**		**II. Based on the power approach**						
**Assumptions and values**		**Assumptions and values**	**Variables**					
			**Age** > **55**		**Secondary education status**		**Underweight**	
Prevalence	28.3%	OR	4.27 [2.82, 6.47]		0.53 [0.38, 0.91]		0.49 [0.24, 0.99]	
CI	95%	CI	95%		95%		95%	
Precision	1.96	Power	80%		80%		80%	
Margin of errors	5%	Ratio	1:1		1:1		1:1	
		% of outcome from not exposed	55.68%		39.54%		14.2%	
		Sample size (*n*)	Kelsey	Fleiss	Kelsey	Fleiss	Kelsey	Fleiss
			82	80	382	378	408	406
Final sample size after adding 10% none response	343	Final sample size adding 10% none response	90.2	88	420	416	**449**	447

The bold values indicate the significant variables.

**Figure 1:**
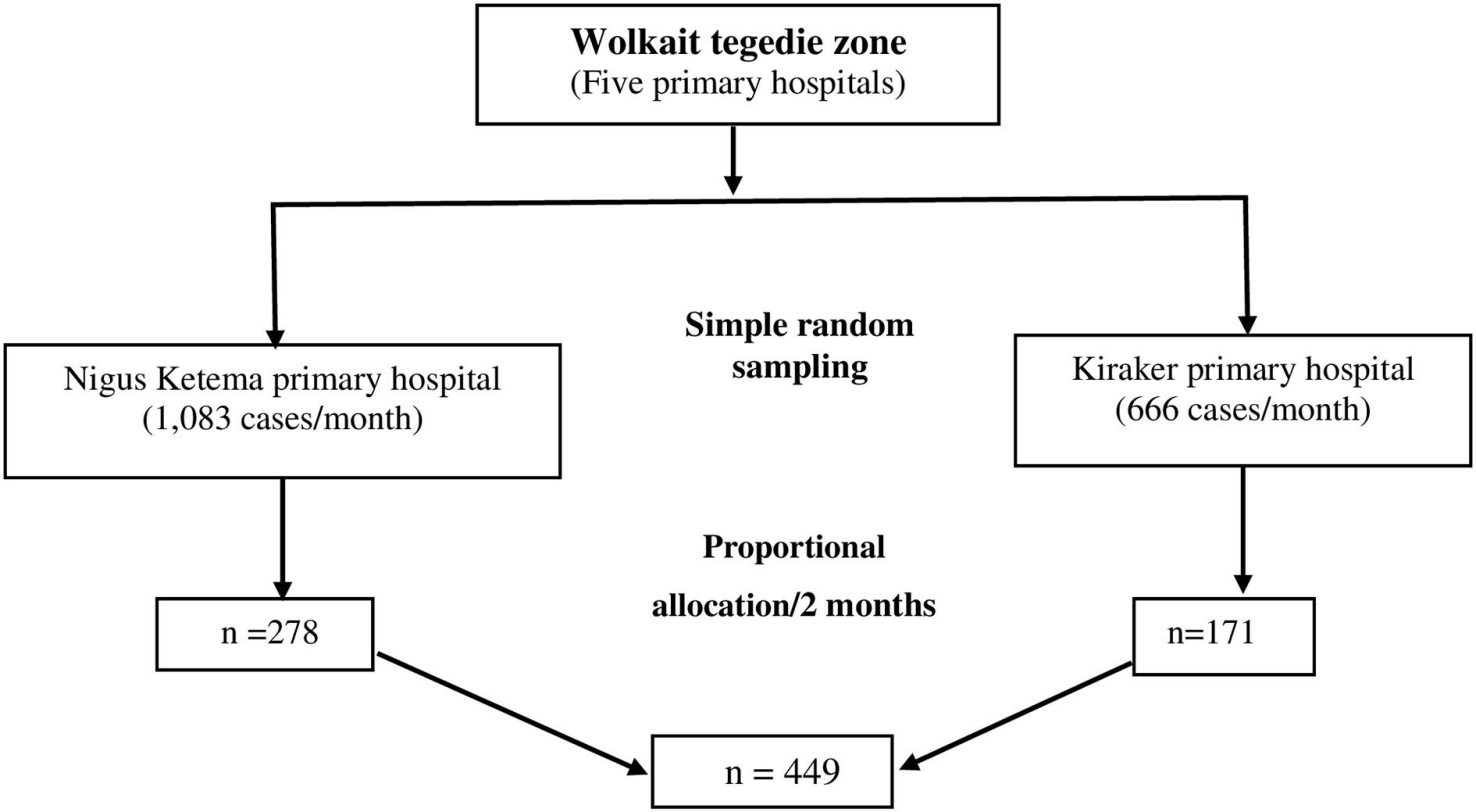
Sampling producers for the study of prevalence and associated factors of hypertension among adult patients evaluated at the OPD in the Kiraker and Nigus Ketema primary hospitals, Northwest Ethiopia, 2021.

Sampling procedures were done for each hospital based on the proportional allocation method. The first two hospitals (Kiraker and Nigus Ketema primary hospitals) were selected using a simple random sampling lottery method from the total of five primary hospitals found in the Wolkait Tegedie zone. The 2 months' (data collection period) expected case fellow status in the OPD of each hospital was 2,166 cases/2 months for the Nigus Ketema primary hospital and 1,334 case/2 months for the Kiraker primary hospital. Therefore, by using the proportional allocation method to select the representative samples from each hospital, 278 from Nigus Ketema primary hospital and 171 from Kiraker primary hospital were selected as the final candidate samples. Then, we calculated the interval for selecting the sample, *k* = ni/Ni (where ni = sample size from each hospital and Ni = number of case fellows in the OPD of each hospital). Then, using systematic random sampling and this k^th^ interval, each hospital sample was selected. Finally, a total sample of 432 patients (96.1%) responded and were included in this study (Figure 1).

### Study variables

The dependent variable of this study was having hypertension. It is defined as when the patient has reported regular use of antihypertensive medication (s) or persistently high blood pressure (systolic blood pressure (SBP) ≥ 130 or diastolic blood pressure (DBP) ≥ 80 mmHg) (14). In contrast, the independent variables included were sociodemographic variables such as age, sex, marital status, educational status, residence, occupation, income status, and body mass index (BMI). Behavioral factors such as cigarette smoking, alcohol drinking, types of oil used, and physical exercise were considered. Moreover, clinical factors such as having a history of diabetes mellitus (DM), family history of hypertension, and family history of DM were included.

### Terms and definitions

#### Hypertension

Defined as when the patient has reported regular use of antihypertensive medication (s) or having an average of two times measurement of high blood pressure (SBP ≥ 130 or DBP ≥ 85 mmHg) (15).

#### BMI

Calculated as weight in kilograms divided by height in square meters and interpreted as underweight (BMI < 18.5), normal (18.5–24.9), overweight (25.0–29.9), and obese (≥ 30.0) (12).

### Data collection procedures

Data were collected using pretested and structured interviewer-administered questionnaire and from a chart review. The data were collected by nurses who have a BSc degree and working in the OPD and supervised by the principal investigator. The interview questionnaire was structured into three sections, namely, sociodemographic characteristics, behavioral factors and medical-related questions, and measurements.

The participants' weight and height were measured and recorded by the interviewers after the interview by following the standard steps. Blood pressure was measured using a standard mercury sphygmomanometer BP cuff with the appropriate cuff size. It was measured two times in a sitting position after reassuring the participant no smoking or caffeine 30 min before measurement and rested for at least 5 min. The second measurement was taken 10 min after the first measurement. Finally, the average of two BP measurements was calculated to determine the BP status of the participant.

### Data quality assurance

The quality of the data was ensured through training of data collectors and supervisors, close supervision, and prompt feedback. The training consisted of instruction on interview techniques as per the prepared tool. We used standard instruments. The accuracy of the measurement instrument was checked before the beginning of each data collection session by the principal investigator and/or data collectors. The data were checked for any inconsistencies, coding errors, out of range, completeness, accuracy, clarity, missing values, and appropriate corrections were made by the principal investigator and the supervisor consistently on a daily basis.

### Data processing and analysis

The survey data were entered into the EPI-INFO version 7 and analyzed using STATA 14 software. Descriptive statistics are presented using texts, graphs, and tables. A binary logistic regression model was used to identify the factors affecting the prevalence of hypertension. Both bivariable and multivariable logistic regression models were carried out. Variables with a *p*-value of < 0.2 in the bivariable analysis were entered into the multivariable analysis. Both the crude odds ratio (COR) and adjusted odds ratio (AOR) with 95% CIs were estimated to show the strength of associations. Finally, a *p*-value of < 0.05 in the multivariable logistic regression analysis was used. For this study, the Hosmer and Lemeshow goodness-of-fit test was used to assess whether the necessary assumptions for the application of multiple logistic regression were fulfilled and whether it was non-significant.

### Ethical considerations

Ethical approval was obtained from the University of Gondar Institutional Ethical Review Board Committee. A support letter was obtained from the University of Gondar Research and Community Service for the respective hospitals, and permission was obtained from two executive directors of the hospitals. Participants were informed about the purpose, objectives, and their right to participate and not to participate in the study. Privacy and confidentiality of the study participants were ensured by not using a personal identifier, and data were collected in private conditions individually. Written informed consent was obtained from the study participants.

## Results

### Background characteristics of study subjects

A total sample of 432 patients was included in this study, with a response rate of 96.1% (432/449). About a quarter (25.23%) of study subjects were found to be aged > 60 years, with a median age of 46 (IQR: 35, 61) years. More than three-fourths (78.24%) of the patients were from rural residences, and 304 (70.37%) patients had a low monthly income. The mean BMI of respondents was 20.63 (± 3.13 SD) kg/m^2^. One-tenth (10.19%) of the participants were overweight. Approximately 55 (12.76%) of patients had preexisting hypertension (Table 2).

**Table 2:** Background characteristics of study subjects in a study of prevalence and associated factors of hypertension among adult patients evaluated at OPD in the Kiraker and Nigus Ketema primary hospitals, Northwest Ethiopia, 2021.

**Variables**	**Categories**	**Frequency (*n*)**	**Percentage (%)**
**Sociodemographic factors**			
Age in years	18–40	125	28.94
	41–60	198	45.83
	>60	109	25.23
Sex	Male	125	29.00
	Female	307	71.00
Marital status	Currently not married	255	58.93
	Currently married	177	41.07
Income status	Low	304	70.37
	Middle	128	29.63
Occupation	Farmer	164	38.14
	Merchant	167	38.84
	Government employ	69	16.05
	Not working	30	6.98
Educational status	Unable to read write	173	40.05
	Unable to read write	195	45.14
	Primary and above	64	14.81
Residence	Urban	94	21.76
	Rural	338	78.24
BMI	Under weight	75	17.36
	Normal	313	72.45
	Over weight	44	10.19
**Behavioral factors**			
Alcohol drink	Non-alcoholic	157	36.68
	Alcoholic	271	63.32
Cigarette smoking	Non-smoker	302	69.91
	Smoker	130	30.09
Physical exercise	Vigorous	105	24.31
	Moderate	175	40.51
	Poor	152	35.19
Types oil used	Liquid oil	169	39.12
	Cruddy oil	233	53.94
	Not oil used	30	6.94
**Clinical variables**			
History of DM	No	40	9.28
	Yes	392	90.72
History of hypertension	No	79	18.29
	Yes	353	81.71
Family history of hypertension	No	56	12.76
	Yes	376	87.24
Family history of DM	No	45	10.42
	Yes	387	89.58

### Prevalence and factors associated with hypertension

The prevalence of hypertension among patients evaluated at the OPD in Kiraker and Nigus Ketema primary hospitals in northwest Ethiopia was 44.91% (95% CI: 40.26%, 49.65%). Of the total of 194 patients with hypertension found in the two hospitals, 124 (63.92%) were newly diagnosed, whereas the remaining 79 (36.08%) were having preexisting hypertension.

**Figure 2:**
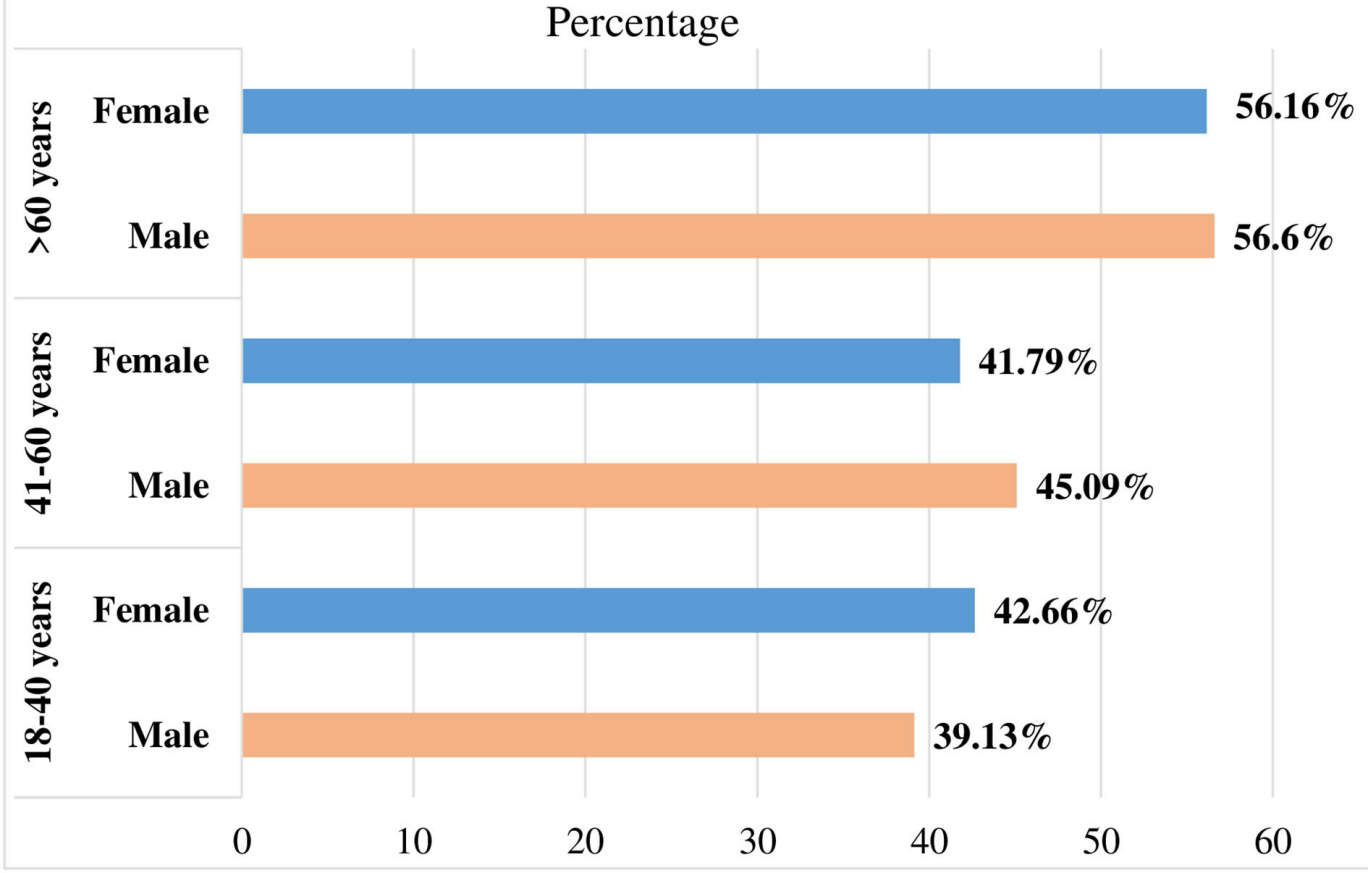
Prevalence of hypertension by sex and age groups among patients in the Kiraker and Nigus Ketema primary hospitals, Northwest Ethiopia.

The prevalence of hypertension was more prevalent among old-age patients (age > 60) (57.8%), patients who consumed crude oil (60.09%), and patients who were doing poor physical exercise (53.95%) but did not have a significant difference between sex (Figure 2).

All variables were analyzed using simple (bivariable) logistic regression analysis to assess the association between each variable having hypertension. Then, variables having a *p*-value of ≤ 0.2 in the simple logistics regression analysis were considered for multiple logistics regression analysis. But variables such as the marital status of the patient with a *p*-value > 0.2 were excluded from multivariable analyses. Moreover, out of those variables considered for multivariate analysis, age of the patient, physical exercise behavior of the patient, types of dietary oil consumption, having a history of DM, and having a family history of hypertension were statistically significantly associated with having hypertension.

Patients >60 years old were likely to have nearly two times higher chance of hypertension compared to patients 18–40 years old [AOR = 1.81; 95% CI: 1.11, 3.20]. The odds of having hypertension among patients who were doing poor physical exercise were 1.74 times higher than in patients who were doing their physical exercise rigorously [AOR = 1.74; 95% CI: 1.01, 3.15]. Patients who consumed cruddy oil were 15.22 times higher to have hypertension compared to those patients who did not consume oil for dietary consumption [AOR = 15.22; 95% CI: 3.86, 60.12].

Patients with a family history of hypertension were 13 times more likely to be hypertensive compared to patients without a family history of hypertension [AOR = 13.02; 95% CI: 3.75, 45.16]. Moreover, patients with a history of DM were eight times more likely to have hypertension compared to those without a DM history [AOR = 8.05; 95% CI: 1.24, 51.49] (Table 3).

**Table 3:** Bivariable and multivariable analyses of factors associated with hypertension among patients in the Kiraker and Nigus Ketema primary hospitals, Northwest Ethiopia, 2021.

**Variables**	**Categories**	**Hypertension**		**COR [95% CI]**	**AOR [95% CI]**
		**No (%) *n* = 194 (44.91)**	**Yes (%) *n* = 238 (55.09)**		
**Sociodemographic and behavioral factors**					
Age in years	18–40	77 (61.6)	48 (38.4)	1.00	1.00
	41–60	115 (58.08)	83 (41.92)	1.16 [0.73, 1.83]	1.01 [0.55, 1.72**]**
	>60	46 (42.2)	63 (57.8)	2.19 [1.30, 3.70]^**^	1.81 [1.11, 3.20]^**^
Sex	Male	69 (55.2)	56 (48.80	1.00	1.00
	Female	168 (54.9)	138 (45.1)	0.99 [0.67, 1.54]	0.64 [0.37, 1.08]
Income status	Low	158 (51.97)	146 (48.03)	1.00	1.00
	Middle	80 (62.5)	48 (37.5)	0.65 [0.43, 0.99]	0.58 [0.35, 1.00]
Occupation	Farmer	82 (50)	82 (50)	1.00	1.00
	Merchant	102 (61.08)	65 (38.92)	0.64 [0.41, 0.98]	0.85 [0.49, 1.47]
	Government employ	41 (59.42)	28 (40.58)	0.68 [0.39, 1.20]	0.84 [0.39, 1.70]
	Not working	13 (43.33)	17 (56.67)	1.31 [0.59, 2.86]	1.78 [0.66, 4.78]
Educational status	Unable to read and write	99(57.23)	74 (42.77)	1.00	1.00
	Able to read and write	108 (55.38)	87 (44.62)	1.08 [0.71, 1.62]	1.05 [0.62, 1.77]
	Primary and above	31 (48.44)	33 (51.56)	1.42 [0.80, 2.53]	0.18 [0.02, 1.83]
Residence	Urban	59 (62.77)	35 (37.23)	1.00	1.00
	Rural	179 (52.96)	159 (47.04)	1.49 [0.94, 2.39]	1.06 [0.59, 1.91]
BMI	Under weight	35 (46.67)	40 (53.33)	1.00	1.00
	Normal	170 (54.31)	143 (45.69)	0.74 [0.44, 1.21]	0.79 [0.43, 1.48]
	Over weight	33 (75)	11 (25)	0.29 [0.13,0.66]	0.56 [0.19, 1.61]
**Behavioral factors**					
Alcohol drink	Non-alcoholic	75 (47.77)	82 (52.23)	1.00	1.00
	Alcoholic	162 (59.78)	109 (40.22)	0.62 [0.41, 0.91]	0.73 [0.44, 1.21]
Cigarette smoking	Non-smoker	172 (56.95)	130 (43.05)	1.00	1.00
	Smoker	66 (50.77)	64 (49.23)	1.28 [0.85, 1.93]	1.31 [0.77, 2.22]
Physical exercise	Vigorous	46 (56.19)	105 (43.81)	1.00	1.00
	Moderate	109 (62.29)	66 (37.71)	1.27 [0.47, 1.27]	0.89 [0.48, 1.65]
	Poor	70 (46.05)	82 (53.95)	1.50 [0.91, 2.47]	1.74 [1.01, 3.15]^*^
Types oil used	Not oil used	25 (83.33)	5 (16.67)	1.00	**1.00**
	Liquid oil	120 (71.01)	49 (28.99)	2.04 [0.74, 5.63]	3.63 [0.89, 14.66]
	Cruddy oil	93 (39.91)	140 (60.09)	7.52 [2.78, 20.36]^**^	15.22 [3.86, 60.12]^**^
**Medical-related conditions**					
History of DM	No	2 (5)	38 (95)	1.00	1.00
	Yes	236 (60.36)	155 (39.64)	28.93 [6.87, 121.64]^***^	8.05 [1.24, 51.49]^**^
Family history of hypertension	No	4 (7.27)	51 (92.73)	1.00	1.00
	Yes	233 (61.97)	143 (38.03)	20.77 [7.35, 58.70]^***^	13.02 [3.75, 45.16]^*^
Family history of DM	No	4 (7.27)	51 (92.73)	1.00	1.00
	Yes	233 (61.97)	143 (38.03)	5.79 [2.72, 12.37]^***^	1.50 [0.46, 4.85]

AOR, adjusted odds ratio; COR, crude odds ratio; CI, confidence interval.

* p-value < 0.05,

** p-value < 0.01,

*** p-value < 0.001.

The bold values indicate the significant variables.

## Discussion

Globally, hypertension is among the leading causes of mortality (14). It is the direct cause of stroke, kidney failure, heart disease, and other complications (14, 16, 17). Even though it is one of the most modifiable risk factors for cardiovascular diseases, the awareness of prevention, treatment, and control of hypertension is extremely low in the developing countries, including Ethiopia. Therefore, this study was used to assess the prevalence of hypertension and possible associated factors among adult patients evaluated at the OPD in Kiraker and Nigus Ketema primary hospitals, northwest Ethiopia. Based on this, the prevalence of hypertension in the study setting was 44.91% (95% CI: 40.26%, 49.65%) and most of the patients were new cases and not aware of being hypertensive. This is in line with a study among older adults in rural Ethiopia (41.9%) (18). But it is higher than a pooled prevalence study all over Ethiopia (21.81%) (7), a hospital-based study in southwest Ethiopia (13.2%) (6), a community-based study in Gondar, Ethiopia (28.3%) (13), Bedele town, southwest Ethiopia (16.9%)(8), Gimbi, Ethiopia (33.5%) (19). These high burdens of hypertension in our findings might be due to stress secondary to the terrifying civil war that happened in the study setting at the time of data collection. The stress secondary to the war can cause hypertension through stimulation of the nervous system to produce large amounts of vasoconstrictor hormones that increase blood pressure (20, 21). Moreover, the availability of trained health coaches in each household might contribute greatly to the prevention of hypertension and continued risk for stroke (22). Different study settings (hospital-based and community-based), the age of the study participants, and sample size might be the additional factors for these discrepancies.

In our study, patients aged >60 years were found to be with nearly two times higher chance of having hypertension compared to patients who were aged 18–40 years. This is supported by a systematic review and meta-analysis (7), a community-based study in Gondar, Ethiopia (13), Gimbi, Ethiopia (19), and a study among older adults in rural Ethiopia (18). This could be due to the biological effect of increased arterial resistance caused by arterial thickening and stiffness that occurs as one gets older (13, 23).

The chance of having hypertension among patients doing poor physical exercise was higher than in patients doing physical exercise rigorously. This is supported by a community-based study in Gondar, Ethiopia (13). Studies showed that regularly performing aerobic exercise significantly decreases blood pressure in patients with essential hypertension (24, 25). Moderate regular exercise is used to increase the elasticity and resistance of arteries (26).

Patients who consumed cruddy oil were considered to have hypertension compared to those patients who did not consume oil for dietary consumption. This is because crude oil ingestion causes abnormality in lipid profile and the risk of incidence of hypertension (27). Crude oil is also known to cause oxidative stress and increased red cell membrane permeability (28).

Patients with a family history of hypertension were more likely to be hypertensive compared to patients without a family history of hypertension. This is supported by a study in Gimbi, Ethiopia (19), southwest Ethiopia (6), a community-based study in Gondar, Ethiopia (13), and the Miyun district of Beijing, China (25). This could be because family members may share genetic factors and mostly exercise similar lifestyles.

Moreover, patients with a history of DM are more likely to have hypertension compared to those without a DM history. This is supported by a systematic review and meta-analysis (7), a community-based study in Gondar, Ethiopia (13), and southwest Ethiopia (6). Thetwo conditions, i.e., hypertension and diabetes, may cause each other and share common risk factors (13, 14, 17).

The strengths of this study come from the use of a relatively large sample size and two district hospitals compared to the previous study conducted in Ethiopia, which makes it representative of populations of study settings. Therefore, it can be generalized to all patients in the Wolkait Tegedie zone during the study period.

This study has some limitations. Since it is a cross-sectional study, causality with explanatory variables cannot be ascertained. We did not include biochemical measurements such as serum glucose level and a 24 h urine sodium concentration.

## Conclusion

There is a relatively high prevalence of hypertension among adult patients in the Kiraker and Nigus Ketema primary hospitals, northwest Ethiopia. Factors such as being older age, having poor physical exercise behavior, having cruddy oil consumption, having diabetes mellitus, and having a family history of hypertension had a positive significant statistical association with being hypertensive. Community-based screening programs for hypertension should be designed and implemented to prevent this silent killer disease. Health education and promotion that focus on healthy nutrition and physical exercise should be delivered. Trained health coaches are necessary per household of each patient with hypertension in the prevention and continued risk for stroke.

## Data availability statement

The raw data supporting the conclusions of this article will be made available by the authors, without undue reservation.

## Ethics statement

The studies involving human participants were reviewed and approved by University of Gondar CMHS ERB. The patients/participants provided their written informed consent to participate in this study.

## Author contributions

Conceptualization, formal analysis, methodology, and supervision: AK, DB, HF, MM, and HA. Data curator and investigation: AK, DA, EM, and DB. Resources: AK, DA, EM, HF, MM, and HA. Software: AK, DB, HF, MM, HA, and EM. Validation: AK, DB, GT, EG, and EM. Visualization and writing original draft: AK, HF, MM, HA, GT, EG, and DB. All authors contributed to the article and approved the submitted version.

## Conflict of interest

The authors declare that the research was conducted in the absence of any commercial or financial relationships that could be construed as a potential conflict of interest.

## Publisher's note

All claims expressed in this article are solely those of the authors and do not necessarily represent those of their affiliated organizations, or those of the publisher, the editors and the reviewers. Any product that may be evaluated in this article, or claim that may be made by its manufacturer, is not guaranteed or endorsed by the publisher.

## Abbreviations

AOR: adjusted odds ratio
COR: crud odds ratio
DBP: diastolic blood pleasure
OPD: outpatient department
SBP: systolic blood pleasure.
